# Dual CD73/A_2A_R blockade modulates the neurotoxic astrocyte phenotype without disrupting core inflammatory signaling

**DOI:** 10.3389/fphar.2026.1778355

**Published:** 2026-04-22

**Authors:** Katarina Mihajlovic, Milorad Dragic, Marija Adzic Bukvic, Teodora Martic, Ivana Stevanovic, Stéphane Vinit, Marêva Bleuzé, Arnaud Mansart, Lucille Adam, Nadezda Nedeljkovic

**Affiliations:** 1 Centre for Translational Neurosciences, Department of General Physiology and Biophysics, Faculty of Biology University of Belgrade, Belgrade, Serbia; 2 Vinča Institute for Nuclear Sciences, University of Belgrade, National Institute of the Republic of Serbia, Belgrade, Serbia; 3 Medical Faculty of the Military Medical Academy, University of Defense, Belgrade, Serbia; 4 Université Paris-Saclay, UVSQ, Inserm U1357, IMPROVE, Versailles, France; 5 Université Paris-Saclay, UVSQ, InsermU1173, Infection et Inflammation (2I), Paris-Saclay, France

**Keywords:** A2A receptor (A2AR), CD73, neuroprotection, neurotoxic reactive astrocyte (nRA) substate, oxidative stress

## Abstract

**Introduction:**

Excessive activation of the adenosine A_2A_ receptor (A_2A_R) contributes to chronic neuroinflammation, in part through spatial coupling with the adenosine-generating enzyme CD73, which enables localized adenosine signaling. Coordinated regulation of *Nt5e* and *Adora2a* across neuropathological conditions supports dual targeting of the CD73/A_2A_R axis to constrain maladaptive inflammatory signaling.

**Methods:**

Primary rat astrocytes were exposed to TNF-α, IL-1α, and C1q (TIC) to induce a neurotoxic reactive astrocyte (nRA) substate. Concomitant pharmacological inhibition of CD73 (APCP, 100 μM) and A_2A_R (istradefylline, 10 μM) was applied. Morphological, redox, inflammatory, and functional outcomes were assessed, including CD73 expression and activity, CD73/A_2A_R spatial proximity, cytokine release, and astrocyte-mediated neurotoxicity.

**Results:**

Dual CD73/A_2A_R blockade attenuated key features of the nRA phenotype, including astrocyte hypertrophy, oxidative stress, and impaired antioxidant capacity. These effects were associated with normalization of CD73 expression and activity, reduced spatial proximity between CD73 and A_2A_R, suppression of IL-1β release and complement- and immune cell-recruiting effector programs (C3, VCAM1), and modulation of redox-sensitive pathways (*Nos2*/NO, NRF2). Notably, IL-6- and TNFα-driven core inflammatory signaling remained preserved. Functionally, dual blockade shifted astrocytes toward a less neurotoxic phenotype, reducing their impact on neuronal Ca^2+^ homeostasis and improving neuronal viability.

**Discussion:**

These findings demonstrate that dual CD73/A_2A_R blockade selectively reconfigures astrocyte inflammatory networks under the tested conditions, without broadly suppressing inflammatory or homeostatic functions at the examined time point. This supports the CD73/A_2A_R axis as a promising therapeutic target for limiting chronic astrocyte-driven neurotoxicity.

## 1 Introduction

Neuroinflammation is an innate immune response of the central nervous system (CNS) to metabolic, ischemic, and mechanical injury or pathogen invasion, with the primary physiological goal of restoring tissue homeostasis (Medzhitov, 2010). This response involves CNS-resident cells, including astrocytes and microglia, vascular endothelial cells and infiltrating immune cells, all of which contribute to the inflammatory environment by producing cytokines, chemokines, and reactive oxygen species (Ransohoff et al., 2012). Among glial cells, astrocytes exhibit remarkable phenotypic and functional heterogeneity, allowing them to either restrain or propagate inflammatory processes depending on the context (Sofroniew, 2015; Hanisch, 2013; Wang et al., 2023). At one end of this spectrum, reactive astrocytes typical of acute brain injury adopt a neuroprotective, pro-resolving phenotype (npRA) that promotes neuronal survival. At the other end, reactive astrocytes prevalent in chronic neurodegeneration acquire a neurotoxic reactive astrocyte phenotype (nRA) characterized by sustained pro-inflammatory signaling, impaired homeostatic support, and harmful effects on neuronal integrity (Parente et al., 2025; Lawrence et al., 2023). Neurotoxic reactive astrocytes, which contribute to the perpetuation of neuroinflammation, are a hallmark of neurodegenerative disorders such as multiple sclerosis (MS), Alzheimer’s disease (AD), and Parkinson’s disease (PD) (Chen et al., 2016).

Among the signaling systems involved in innate immunity and neuroinflammation, purinergic signaling occupies a central regulatory role (Aminin and Illes, 2021). It is hierarchically organized, with upstream extracellular nucleotide metabolism controlling the balance between ATP, AMP, and adenosine, and downstream receptor activation translating these fluctuations into cell-specific functional responses. Within this framework, adenosine occupies a pivotal modulatory role (Boison et al., 2010), as its effects depend on extracellular concentration and adenosine receptor (ARs) availability, allowing it to support homeostatic and neuroprotective functions or, under certain conditions, to promote neurotoxic astrocyte responses (Guo et al., 2026; Haskó and Cronstein, 2013). Under physiological conditions, extracellular adenosine is maintained at nanomolar levels through equilibrative nucleoside transporters (ENTs) (Newby et al., 1985), whereas acute metabolic stress rapidly elevates adenosine to micromolar concentrations (Melani et al., 2012). In contrast, during chronic neuroinflammation, sustained ATP release from stressed or dying cells represents the predominant source of extracellular adenosine (Shigetomi et al., 2024). At micromolar concentrations, extracellular ATP functions as a danger-associated molecular pattern, activating P2X7 receptors and promoting NLRP3 inflammasome assembly and IL-1β maturation, thereby amplifying inflammation (Melani et al., 2012; Cicko et al., 2018). This pro-inflammatory ATP-driven signaling is counterbalanced by extracellular nucleotide hydrolysis mediated by the ectonucleotidases CD39 and CD73, whereby CD39 converts ATP and ADP to AMP, and CD73, predominantly expressed by reactive astrocytes, subsequently hydrolyzes AMP to adenosine (Zimmermann, 2000; Zimmermann et al., 2012). Thus, the coordinated activity of CD39 and CD73 determines the extracellular ATP/adenosine balance, shaping the magnitude and temporal dynamics of neuroinflammation and influencing microglial and astrocyte phenotypic fate (Antonioli et al., 2013; Illes et al., 2020).

Adenosine mediates its effects through 4 G protein-coupled receptors (A_1_R, A_2A_R, A_2B_R, and A_3_R), all functionally coupled to adenylyl cyclase (AC) activity (Borea et al., 2018). High-affinity A_1_R and A_3_R couple to Gi proteins, which inhibit AC and suppress cAMP levels, whereas A_2A_R and A_2B_R couple to Gs proteins, which stimulate AC and increase cAMP production. Under physiological conditions, adenosine signaling is dominated by A_1_R activation, supporting homeostasis and neuroprotection. In contrast, during neuroinflammation, elevated extracellular adenosine shifts signaling toward A_2A_R and A_2B_R, promoting glial activation and inflammatory mediator release (Orr et al., 2009; Cunha, 2016; Borroto-Escuela et al., 2018). Accumulating evidence implicates excessive A_2A_R activation and maladaptive receptor complexes with CD73 in a range of neurological disorders (Borroto-Escuela et al., 2018; Nedeljkovic, 2019; Dragić et al., 2021). Neuronal A_2A_R overexpression accelerates synaptic dysfunction, while pathological upregulation of A_2A_R in astrocytes disrupts core homeostatic functions and promotes a pro-inflammatory, neurotoxic phenotype (Carvalho et al., 2019; Moreira-de-Sá et al., 2021; Launay et al., 2025). Notably, excessive A_2A_R activation is sufficient to induce brain dysfunction even in the absence of overt injury, whereas normalization of A_2A_R activity may attenuate neuroinflammation and confer neuroprotection in EAE (Dragić et al., 2021). Clinical approval of the selective A_2A_R antagonist istradefylline (Nourianz®) further underscores the translational relevance of targeting this pathway. Importantly, spatial coupling of CD73 with A_2A_R in inflammatory conditions enables localized adenosine generation and receptor activation within same microdomains, establishing a self-perpetuating signaling loop that sustains chronic glial reactivity and converts adenosine from an immunosuppressive to a pro-inflammatory mediator (Gomes et al., 2011; Ye et al., 2023; Matos et al., 2012). Consistent with this functional integration, coordinated regulation of *Nt5e* and *Adora2a* expression has been reported across multiple neuropathological conditions (Aug et al., 2013; Ng et al., 2015; Barros-Barbosa et al., 2016), supporting their joint contribution to neuroinflammation (Cunha, 2016; Nedeljkovic, 2019). These observations provide a strong rationale for dual targeting of the CD73/A_2A_R axis, rather than inhibition of either component alone, as a therapeutic strategy to constrain chronic inflammatory signaling.

In the present study, we directly tested this concept by examining how concomitant inhibition of CD73 and A_2A_R modulates astrocyte reactivity *in vitro*. A neurotoxic reactive astrocyte (nRA) substate was induced by exposing primary cortical astrocytes to a combination of microglia-derived cytokines, tumor necrosis factor-α (TNF-α), interleukin-1α (IL-1α), and complement component C1q, collectively termed TIC (Liddelow et al., 2017), whereas dual CD73/A_2A_R blockade was effectuated using the CD73 inhibitor α,β-methylene diphosphate (APCP) and the selective A_2A_R antagonist istradefylline. Our findings demonstrate that dual CD73/A_2A_R blockade markedly attenuates TIC-induced nRA features by normalizing astrocyte morphology, oxidative stress, and intrinsic antioxidant defenses, while selectively suppressing complement- and immune cell-recruiting gene programs and preserving core cytokine-driven inflammatory signaling. Collectively, these data provide a rationale for targeting the CD73/A_2A_R axis as a means of limiting neurotoxic astrocyte reactivity without broadly disrupting inflammatory signaling.

## 2 Materials and methods

### 2.1 Animals

A total of 28 male rat pups of the Wistar strain from the institutional animal facility (University of Belgrade, Faculty of Biology) were used in the study. All animal procedures were approved by the Ethical committee of Faculty of Biology and performed under permit issued by Veterinary Administration of the Ministry of Agriculture, Forestry and Water Management of the Republic of Serbia (authorization reference number 323-07-02890/2023-05).

### 2.2 Primary astrocyte cultures

Primary astrocyte cultures were prepared from cortical tissue of individual neonatal rat pups at postnatal day 1–2 (P1-2) obtained from different litters, as previously described (Mihajlovic et al., 2023). Briefly, cortices were dissected in cold sterile phosphate-buffered saline (PBS), mechanically dissociated in Dulbecco’s Modified Eagle Medium (DMEM; Sigma-Aldrich, United States), centrifuged three times at 500 × g for 5 min, and passed sequentially through 21G and 23G needles. The resulting cell suspension was resuspended in DMEM+ (DMEM supplemented with 10% fetal bovine serum (FBS; Gibco, United States), 1 mM sodium pyruvate (Sigma-Aldrich, United States), and antibiotics (100 IU/mL penicillin, 100 μg/mL streptomycin; Gibco, United States)), and maintained at 37 °C in a humidified atmosphere of 5% CO_2_/95% air.

Culture media were replaced on days 2 and 4 to remove floating microglia and oligodendrocyte precursor cells. On day 5, adherent cells were detached using 0.25% trypsin and 0.02% EDTA (both from Sigma-Aldrich, United States), reseeded in fresh DMEM+, and maintained with medium changes every other day to favor astrocyte proliferation. Gentle washing during each change further reduced non-astrocytic contamination. Confluent cells were trypsinized, counted, and plated for experiments on 35-mm or 60-mm Petri dishes, multi-well plates (24-, 12-, or 6-well), or poly-L-lysine–coated coverslips (15-mm diameter) at a density of 20,000 cells/cm^2^. This procedure routinely yields primary astrocyte cultures with <2% non-astrocytic cells. All experiments were performed 15–25 days after isolation.

### 2.3 Induction of neurotoxic astrocyte phenotype and dual CD73/A_2A_R blockade

Near-confluent primary astrocyte cultures were stimulated with a combination of human recombinant cytokines (TIC) comprising TNFα (30 ng/mL; R&D Systems, 210-TA/CF), IL-1α (3 ng/mL; R&D Systems, 200-LA-010/CF), and C1q (400 ng/mL; R&D Systems, 9134-TN-050), dissolved in DMEM + or DMEM + without sodium pyruvate (for oxidative stress measurements). In preliminary experiments, cells were stimulated with TIC for 8–32 h and neurotoxic reactive phenotype was assessed based on the expression profiles of key inflammatory genes. Based on the expression dynamic the 24 h time point was selected for subsequent experiments (TIC-stimulated culture).

Dual CD73/A_2A_R blockade was achieved using the established CD73 inhibitor α,β-methylene adenosine 5′-diphosphate (APCP; Sigma-Aldrich, M3763) at a concentration of 100 μM and the selective A_2A_R antagonist istradefylline (Sigma-Aldrich, SML0422) at 10 μM. Concentrations were chosen within the range commonly used to achieve robust functional inhibition of CD73 and A_2A_R signaling *in vitro*, while maintaining cellular viability (Mihajlovic et al., 2023). To evaluate the effects of dual CD73/A_2A_R blockade, astrocyte cultures were first stimulated with TIC for 16 h, followed by concomitant treatment with 100 μM APCP and 10 μM istradefylline for an additional 8 h. The resulting cultures (Dual blockade) were directly compared with TIC-stimulated astrocytes.

### 2.4 Primary neuronal culture

Primary cerebellar granule neurons (CGN) were prepared from neonatal pups at P6–P7, according to protocol previously described (Tehran and Pirazzini, 2018). After decapitation, brains were rapidly removed and transferred into ice-cold DMEM supplemented with 0.1 mg/mL streptomycin and 100 IU/mL penicillin (Gibco, ThermoFisher Scientific). The meninges were carefully removed under a stereomicroscope, and the cerebellum was dissected from the remaining brain tissue. The tissue was enzymatically digested with 1 mg/mL trypsin in DMEM with antibiotics, at 37 °C for 10 min. Enzymatic digestion was terminated by washing the tissue three times with NeuroBasal-A medium (Gibco) containing 2% FBS. The partially dissociated tissue was mechanically triturated in 200 μL of the same medium using a 200 μL pipette tip until a homogeneous cell suspension was obtained. The suspension was transferred to a 15 mL conical tube, diluted with 1 mL medium, and centrifuged at 400 *g* for 4 min at room temperature. The supernatant was discarded, and the pellet was gently resuspended in 200 μL medium, followed by addition of 900 μL medium and a second centrifugation under identical conditions. After removal of the supernatant, the final pellet was resuspended in growth medium consisting of NeuroBasal-A supplemented with 2% FBS, 1% GlutaMAX (Gibco, ThermoFisher Scientific), 1% B27 supplement, 25 mM NaCl, and 10 μg/mL gentamicin (Sigma), with osmolality adjusted to ∼320 mOsm/kg.

Cells were plated onto 10 mm glass coverslips pre-coated with 50 μg/mL poly-L-ornithine (PLO) and placed in 24-well plates. The cell suspension obtained from one cerebellum was evenly distributed across 24 wells. On day *in vitro* 1 (DIV1), the medium was completely replaced with fresh growth medium and the cells were treated on DIV2-3 Cultures were maintained at 37 °C in a humidified incubator with 5% CO_2_ to ensure physiological pH stability and optimal culture conditions. The highly enriched CGN cultures were used for subsequent Ca^2+^ imaging and propidium iodide staining.

### 2.5 Assessment of intracellular Ca^2+^ dynamics and neuronal cell death

The ability of dual CD73/A_2A_R blockade to attenuate the neurotoxic astrocyte (nRA) phenotype was assessed in primary cerebellar granule neurons (CGNs) after 24 h exposure to conditioned media obtained from control (control-ACM), TIC-stimulated (TIC-ACM), or dual blockade-treated (dual blocker-ACM) astrocyte cultures. For Ca^2+^ imaging CGNs were loaded with 5 µM Fluo-4 AM (Thermo Fisher Scientific) in extracellular solution (ECS: 140 mM NaCl, 5 mM KCl, 2 mM CaCl_2_, 1 mM MgCl_2_, 10 mM HEPES, 10 mM glucose; pH 7.4; ∼320 mOsm) for 30 min at 37 °C, followed by a 10 min wash in ECS containing propidium iodide (PI; 2 μg/mL). Imaging was performed on an AxioObserver A1 inverted microscope (Zeiss) equipped with a 25×/0.8 NA water-immersion objective and an EM512 CCD camera (Photometrics), controlled by VisiView software. Images were acquired at 1 Hz. Fluo-4 and PI were recorded using standard FITC and TRITC filter sets, respectively, with xenon lamp illumination. Cells were continuously perfused with ECS (4 mL/min). Baseline fluorescence was recorded for 100 s, after which 80 μL TIC-ACM was applied directly to the chamber and perfusion paused for 120 s. Neuronal viability and responsiveness were confirmed by depolarization with high-K^+^ ECS (50 mM K^+^). Fluorescence intensity was quantified in ImageJ from somatic ROIs and expressed as F/F_0_ (baseline-normalized) using a custom MATLAB script. PI and brightfield images were acquired to verify membrane integrity and morphology and the representative images are shown in Supplementary Figure S3.

For independent assessment of neuronal death, CGNs were exposed to TIC-ACM for 24 h, incubated with PI (2 μg/mL, 10 min, 37 °C), and imaged live under identical acquisition settings across groups. Corresponding brightfield images were acquired to confirm comparable cell density. PI-positive nuclei were quantified in ImageJ using the Analyze Particles function with consistent thresholding across all images. Data were expressed as the mean number of PI-positive nuclei normalized to the total number of cells, calculated from 10 non-overlapping fields per group (±SEM).

### 2.6 Isolation of cell lysates and separation of culture media

Culture media from near confluent cultures (control, TIC-stimulated and dual blockade) were removed, centrifuged for 10 min at 500 × g to pellet residual cells and frozen at −80 °C until use. Remaining cells were washed twice, scraped in ice-cold PBS buffer, and centrifuged at 10,000 × g for 15 min at 4 °C. The cell lysate containing supernatant was separated and frozen at −80 °C until use. Protein content was determined using the Pierce™ BCA protein assay kit (Thermo Fisher Scientific, Waltham, MA, United States of America).

### 2.7 Crystal violet (CV) assay

Cells were seeded in 96-well PLL pre-coated plates. After treatment, cells were fixed with 4% paraformaldehyde (PFA) for 15 min at 4 °C, rinsed with PBS, and stained with 0.5% crystal violet solution (Cat. No. 32675, Fluka Analytical, Switzerland) for 20 min at RT. Excess dye was removed by rinsing plates several times with tap water, followed by air drying at 45 °C for 1 h. The retained dye was solubilized with 50 µL of glacial acetic acid per well. Absorbance was measured at 570 nm (A_570_) using a BioTek Epoch Microplate Spectrophotometer (BioTek Instruments, United States). Data are presented as mean relative absorbance (%) ± SEM, obtained from three independent astrocyte culture preparations per experimental group, with each measurement performed in triplicate.

### 2.8 MTT assay

Cells were cultured in 24-well plates and, after treatment, incubated with 3-(4,5-dimethylthiazol-2-yl)-2,5-diphenyltetrazolium bromide (MTT; 0.5 mg/mL; M5655, Sigma-Aldrich, United States) for 30 min at 37 °C. Formazan crystals formed by metabolically active cells were dissolved in 720 µL of dimethyl sulfoxide (DMSO). Aliquots (200 µL per sample, in triplicate) were transferred to a 96-well plate, and absorbance was measured at 570 nm using a BioTek Epoch Microplate Spectrophotometer (BioTek Instruments, United States) and results are expressed as mean relative absorbance (%) ± SEM, from two independent culture preparations for each experimental groups, with each measurement performed in quadruplicate.

### 2.9 Bright field microscopy

For bright-field imaging, astrocytes were seeded in 35-mm Petri dishes. Images from three randomly selected fields per dish were acquired using an Axio Observer A1 inverted microscope (Carl Zeiss GmbH, Jena, Germany) equipped with an A-Plan ×10 objective and an EM512 CCD camera system (Evolve, Photometrics, United States).

Astrocyte morphology was quantified on bright-field micrographs using *ImageJ* software. Cell area, perimeter, aspect ratio, circularity, and solidity were calculated from automatically outlined cell bodies to assess changes in astrocyte size, shape anisotropy, and contour complexity. Mean area (μm^2^), perimeter (μm) and shape descriptor values were expressed as a mean ± SEM, obtained from ≥10 cells in 10 random and non-overlapping microscopic fields from three culture preparations.

### 2.10 Malachite green enzyme assay

Cells were cultured in 24-well plates and, after treatment, washed three times with phosphate-free medium containing 117 mM NaCl, 5.3 mM KCl, 1.8 mM MgCl_2_, 26 mM NaHCO_3_, and 10 mM glucose (pH 7.4). The 5′-phosphohydrolase activity catalyzed by CD73 was determined by quantifying inorganic phosphate (Pi) released into the medium using the malachite green colorimetric assay, originally described by Baykov et al. (1988) and modified as described (Adzic and Nedeljkovic, 2018). Briefly, the enzymatic reaction was initiated by adding AMP to a final concentration of 1 mM in phosphate-free medium. After 30 min incubation at 37 °C, the reaction was stopped by transferring the medium into tubes containing ice-cold 3 M perchloric acid (PCA). Cells were lysed in 100 µL of RIPA buffer, and total protein concentration was determined using the Pierce™ BCA Protein Assay Kit (Thermo Fisher Scientific, United States).

Aliquots (80 µL) of each sample were transferred to 96-well plates and mixed with 20 µL of freshly prepared malachite green reagent (0.1% malachite green in 20% H_2_SO_4_, 7.5% ammonium molybdate, and 11% Tween 20; mixed in a 10 : 2.5: 0.2 ratio). After 30 min incubation at RT, absorbance was measured at 620 nm using a microplate reader. The concentration of liberated Pi was calculated from a standard curve generated with KH_2_PO_4_ standards. Control reactions were performed in parallel to correct for non-enzymatic hydrolysis of AMP, by omitting AMP during incubation and adding it only after PCA addition. The results are expressed as mean CD73 activity (µmol Pi mg-1 protein min-1) ± SEM, from four different culture preparations, with each measurement performed in quadruplicate.

### 2.11 Nitric oxide determination

Cells were cultured in 6-well plates. Nitric oxide (NO) release into the culture medium was quantified using the Griess colorimetric assay, based on cadmium-mediated reduction of nitrates to nitrites (Navarro-Gonzálvez et al., 1998). Briefly, 50 µL of each medium sample was mixed with Griess reagent [1.5% sulfanilamide in 1 M HCl and 0.15% N-(1-naphthyl) ethylenediamine dihydrochloride in distilled water] and incubated for 10 min at room temperature (RT). Absorbance was measured at 492 nm using a microplate spectrophotometer. Nitrite concentrations were calculated from a standard curve generated with sodium nitrite (NaNO_2_). Results are expressed as mean NO concentration (µmol/L) ± SEM, from five different culture media preparations, with each measurement performed in triplicate.

### 2.12 Determination of malondialdehyde (MDA)

Cells were cultured in 6-well plates. Levels of malondialdehyde (MDA), an index of lipid peroxidation, were determined spectrophotometrically in the culture medium using the thiobarbituric acid reactive substances (TBARS) assay (Villacara et al., 1989). Medium samples (200 µL) were incubated with 400 µL of TBA reagent [15% trichloroacetic acid and 0.375% TBA in aqueous solution, pH 3.5] for 5 min at 95 °C. Samples were cooled, centrifuged at 3,000 × g for 1 min, and 300 µL of the supernatant was transferred, in duplicate, to a 96-well plate. Absorbance was measured at 532 nm using a microplate spectrophotometer. MDA concentrations are normalized to total protein content (µmol/L) ± SEM, from three different culture media preparations, with each measurement performed in triplicate.

### 2.13 Determination of glutathione (GSH)

Astrocytes were seeded in 6-well plates and treated as described. Total glutathione (GSH) levels were quantified in cell lysates using the DTNB-glutathione reductase recycling assay, in which oxidized glutathione (GSSG) is reduced to GSH in the presence of NADPH (Anderson, 1985). Aliquots (25 µL) of cell lysates were mixed with 700 µL NADH buffer, 100 µL DTNB buffer, and 175 µL deionized water. The reaction was initiated by adding 10 µL of glutathione reductase, and the formation of 5-thio-2-nitrobenzoic acid (TNB), proportional to GSH concentration, was monitored spectrophotometrically at 412 nm. Results are expressed as mean GSH concentration (nmol/mg) ±SEM, from five different culture preparations.

### 2.14 Determination of total sulfhydryl content (SH-)

Total sulfhydryl content was determined based on the reaction of DTNB (Ellman’s reagent) with aliphatic thiol compounds at pH 9, producing 1 mole of *p*-nitrophenol anion per mole of thiol. The reaction mixture contained 900 μL K_2_HPO_4_ buffer, 20 μL DTNB and 50 μL sample. After incubation for 25 min at RT, the absorbance was measured at 412 nm. Results are expressed as mean SH^−^ concentration (nmol mg^-1^ protein) ± SEM, from five different culture preparations, with each measurement performed in triplicate.

### 2.15 Determination of superoxide dismutase (SOD) activity

Astrocytes were cultured in 6-well plates and treated as described. Cells were harvested and lysed, and total superoxide dismutase (tSOD) activity, including cytosolic Cu/ZnSOD and mitochondrial MnSOD isoforms, was determined using the epinephrine auto-oxidation method (Sun and Zigman, 1978). Briefly, 50 µL of each lysate sample was added to a reaction mixture containing 50 mM carbonate buffer (pH 10.2), 1 mM EDTA, and 10 mM epinephrine. To selectively determine MnSOD activity, potassium cyanide (KCN) was added to inhibit Cu/ZnSOD, whereas Cu/ZnSOD activity was calculated as the difference between total and MnSOD activities. Absorbance was measured at 480 nm using a spectrophotometer. Enzyme activity was expressed as units per milligram of protein (U/mg), with one unit defined as the amount of enzyme producing 50% inhibition of epinephrine auto-oxidation, ±SEM, from five different culture preparations, with each measurement performed in triplicate.

### 2.16 Determination of catalase activity

Catalase activity in cell lysates was determined spectrophotometrically by measuring the yellow complex formed between ammonium molybdate and residual hydrogen peroxide (H_2_O_2_) (Góth, 1991). Briefly, 100 µL of cell lysate was incubated with 65 μmol/mL H_2_O_2_ prepared in 6 mM sodium–potassium phosphate buffer (pH 7.2). After 1 min, the reaction was stopped by adding 32.4 mM ammonium molybdate, and absorbance was measured at 405 nm using a spectrophotometer. Catalase activity was expressed as units per milligram of protein (U/mg), where one unit corresponds to the amount of enzyme decomposing 1 µmol of H_2_O_2_ per minute, ±SEM, from three different culture preparations, with each measurement performed in triplicate.

### 2.17 Quantitative real-time PCR

For quantitative RT-PCR (qRT-PCR) analysis, cells were grown on 35-mm Petri dishes, collected in NZYzol (NYZtech, Lisbon, Portugal) and frozen at −80 °C. Total RNA was isolated in phenol/guanidinium solution and the concentration and purity of RNA were determined by measuring 260/280 nm and 260/230 nm ratios, respectively (BioTek Epoch Microplate Spectrophotometer). A volume equivalent of 1 μg of total RNA was transcribed into cDNA using the High Capacity cDNA Reverse Transcription Kit (Applied Biosystems, Foster City, CA, United States). The PCR mixture contained 2 μL cDNA (at a final concentration of 2 ng/μL), 2 μL RNase-free water (UltraPure, Invitrogen, Germany), 0.5 μL primer (100 pmol/μL) and 5 μL QTM SYBR Green PCR Master Mix (Applied Biosystems, Foster City, CA, United States). The PCR reaction included 10 min of enzyme activation at 95 °C, 40 cycles of 15 s denaturation at 95 °C, 30 s annealing at 64 °C, 30 s amplification at 72 °C and 5 s fluorescence measurements at 72 °C (QuantStudioTM 3 Real-Time PCR System, Applied Biosystems, Foster City, CA, United States). *Gapdh* was used as an internal control and quantification was performed by the 2^−ΔCt^ method. Samples from each culture (n ≥ 4) were run in duplicate. Primers sequences are listed in Table 1.

**TABLE 1:** List of primers.

Target gene	Forward	Reverse
*Adora2a*	TGC​AGA​ACG​TCA​CCA​ACT​TC	CAA​AAC​AGG​CGA​AGA​AGA​GG
*Apoe*	CCC​CGG​AGG​CTA​AGG​AGT​T	TCT​GTC​ACC​TCC​AGC​TCT​CC
*C3*	GCG​GTA​CTA​CCA​GAC​CAT​CG	CTT​CTG​GCA​CGA​CCT​TCA​GT
*Ccl2*	CAG​GTC​TCT​GTC​ACG​CTT​CTG	CTC​CAG​CCG​ACT​CAT​TGG​G
*Cebpb*	CCACGTCGTCGTCGTCC	GTA​CTC​GTC​GCT​CAG​CTT​GT
*Cxcl16*	CAG​CCA​GGG​TGA​AGT​GAA​AGC	TCA​CAG​TAG​CAA​CTT​CCA​GCG
*Gapdh*	CAA​CTC​CCT​CAA​GAT​TGT​CAG​CAA	GGC​ATG​GAC​TGT​GGT​CAT​GA
*Gfap*	CGG​CTC​TGA​GAG​AGA​TTC​GC	GGT​CTG​CAA​ACT​TGG​ACC​GA
*Il1b*	AAA​CAG​CAA​TGG​TCG​GGA​CA	GTC​CTG​GGG​AAG​GCA​TTA​GG
*Il6*	GCC​CAC​CAG​GAA​CGA​AAG​T	GGC​AAC​TGG​CTG​GAA​GTC​TC
*Il10*	GCT​CAG​CAC​TGC​TAT​GTT​GC	GTC​TGG​CTG​ACT​GGG​AAG​TG
*Lcn2*	GGA​TCA​GAA​CAT​TCG​TTC​CA	ATG​GCA​AAC​TGG​TCG​TAG​TC
*Lif*	TCA​ACT​GGC​TCA​ACT​CAA​CG	AAA​GGT​GGG​AAA​TCC​GTC​AT
*Nfe2l2*	GAC​TTG​GAA​TTG​CCA​CCG​C	CCT​GTT​CCT​TCT​GGA​GTT​GCT
*Nos2*	ACA​CAG​TGT​CGC​TGG​TTT​GA	AAC​TCT​GCT​GTT​CTC​CGT​GG
*Nt5e*	CAA​ATC​TGC​CTC​TGG​AAA​GC	ACC​TTC​CAG​AAG​GAC​CCT​GT
*Prkacb*	GGT​GGG​CAT​TGG​GTG​TAC​TG	GAA​CTG​AAG​TGC​GAC​GGG​AA
*Rela*	AGC​ATG​TAC​AGA​TTC​TGG​GGA​G	AGA​GCC​GAC​TAT​CGT​ACA​GGG
*S100a10*	GTA​CCC​ACA​CCT​TGA​TGC​GT	CGA​AAG​CTC​CTC​TGT​CAT​TGG
*Serping1*	AGG​CTA​ACT​GGC​TTC​GTA​GG	CGG​GAG​CCA​TCT​CTT​TCA​GG
*Stat3*	TGT​GAC​ACC​AAC​GAC​CTG​C	ACA​CTC​CGA​GGT​CAG​ATC​CA
*Tnf*	CTC​CCA​GAA​AAG​CAA​GCA​AC	CGA​GCA​GGA​ATG​AGA​AGA​GG
*Vcam1*	GGG​GAT​TCC​GTT​GTT​CTG​AC	TCT​CCA​GTT​TCC​TTC​GCT​GAC

### 2.18 Immunocytochemistry and confocal microscopy

Cells were seeded onto poly-L-lysine–coated 15 mm coverslips (50 μg/mL; Sigma-Aldrich, United States) placed in 12-well plates. After treatment, cells were rinsed with PBS and fixed with 4% paraformaldehyde (PFA) for 20 min at room temperature (RT). For intracellular staining, cells were permeabilized with 0.1% Triton X-100 in PBS for 15 min at RT, followed by blocking with 5% bovine serum albumin (BSA) in PBS for 1 h at RT. Cells were then incubated with primary antibodies overnight at 4 °C, washed three times with PBS, and incubated with fluorophore-conjugated secondary antibodies for 2 h at RT. Nuclei were counterstained with DAPI (D1306; Invitrogen, United States) for 15 min, followed by five PBS washes (5 × 5 min). Coverslips were mounted in MOWIOL mounting medium (Sigma-Aldrich, United States). Antibodies used in the study are listed in Table 2.

**TABLE 2:** List of primary and secondary antibodies used in the study.

Antibody selectivity	Source and clonality	Dilution/Application	Manufacturer/RRID
A_2A_R	Rabbit, *pc*	1:100 IF	Invitrogen PA1-042 AB_2257858
CD73	Guinea pig, *pc*	1:100 IF	ectonucleotidases-ab.com Cat# rNu-4CI4; rNu-5C(I4,I5); rNu-6C(I4,I5)
GFAP	Chicken, *pc*	1:200, IF	Novus Cat# NBP1-05198 AB_1556315
Alexa Fluor 488 Chicken IgY (H + L)	goat, *pc*	1:200 IF	Invitrogen, A-11039 AB_ 2534096
Alexa Fluor 647 Chicken IgY (H + L)	goat, *pc*	1:200 IF	Invitrogen, A-21449 AB_2535866
Alexa Fluor 555 Rabbit IgG (H + L)	Donkey, *pc*	1:200 IF	Invitrogen, A-31572 AB_141784

Fluorescence images were acquired using a confocal laser-scanning microscope (LSM 510; Carl Zeiss GmbH, Jena, Germany) equipped with Ar multi-line (457, 478, 488, 514 nm) and HeNe (543 nm) lasers, a ×40 oil-immersion differential interference contrast (DIC) objective, and a monochrome AxioCam ICm1 camera. A digital zoom factor of 2× was applied. For each experimental condition, ten randomly selected fields containing comparable cell numbers were analyzed using ImageJ software (NIH, United States). Fluorescence intensity was quantified as background-corrected integrated density and expressed in arbitrary units (a.u.), measured in >10 random, non-overlapping microscopic fields obtained from two independent culture preparations per experimental group. These data were used to calculate the Pearson correlation coefficient (PCC) and Manders’ colocalization coefficients (MCC). PCC values range from −1 to +1 and reflect the degree of linear correlation between pixel intensity values of two signals, independent of their absolute intensities. MCC values range from 0 to 1 and quantify the fraction of one signal that spatially overlaps with the other, providing a direct measure of colocalization. MCC1 represents the fraction of signal 1 overlapping with signal 2, while MCC2 represents the fraction of signal 2 overlapping with signal 1 (Manders et al., 1993; Dunn et al., 2011).

### 2.19 Enzyme-linked immunosorbent assay (ELISA)

Culture media from control, TIC cultures and cultures treated with dual blockade grown in 60-mm Petri dishes were collected and stored at −80 °C until analysis. Concentrations of selected target proteins were quantified using commercially available ELISA kits according to the manufacturers’ instructions. The following assays were used: Rat Complement C3 ELISA Kit (E-EL-R0250, RRID: SCR_025982), Rat Neutrophil Gelatinase–Associated Lipocalin (NGAL) ELISA Kit (E-EL-R3055, RRID:SCR_025982), and Rat Vascular Cell Adhesion Molecule-1 (VCAM-1/CD106) ELISA Kit (E-EL-R1061; RRID:SCR_025982), all from Elabscience, China. For quantification of TNF-α, IL-10, IFN-γ, CXCL1, CCL2, GM-CSF, IL-12p70, IL-1β, IL-17A and IL-6, the LEGENDplex™ Rat Inflammation Panel 13-plex (BioLegend, Canada, RRID: SCR_001134) was used, following the manufacturer’s protocol. Results are expressed as mean cytokine concentration (pg/μl or ng/μl) ± SEM, based on three independent culture preparations for each experimental group, with each preparation analyzed in duplicate.

### 2.20 Network analysis

Network analysis was performed using the Cytoscape (version 3.10.4). Log2 fold-change, (log2FC) and *p* values of total of 22 identified genes (nodes) were used as input, maintaining identical parameters across all experimental groups. Visualization of connections (edges) between nodes was done using STRING representation. Direction of gene expression was represented with continuous color, where color intensity represents level of the change. Red shades indicate upregulation (positive log2FC value), blue shades represent downregulation (negative log2FC values), while white represents lack of change. Border width is positively associated with statistical significance of gene expression.

### 2.21 Data analysis

All statistical analyses and graphical representations were performed using GraphPad Prism version 9 (GraphPad Software, United States). Data normality was assessed using the Shapiro–Wilk test. For comparisons between two normally distributed groups, a two-tailed unpaired Student’s t-test was applied. For comparisons involving more than two groups with normally distributed data, one-way ANOVA followed by Tukey’s *post hoc* test was used. When data were not normally distributed, the non-parametric Mann–Whitney U test was applied. A p-value <0.05 was considered statistically significant. Data are presented as mean ± standard error of the mean (SEM) from *n* independent cultures.

## 3 Results

### 3.1 Validation of the TIC-induced reactive astrocyte model

**FIGURE 1:**
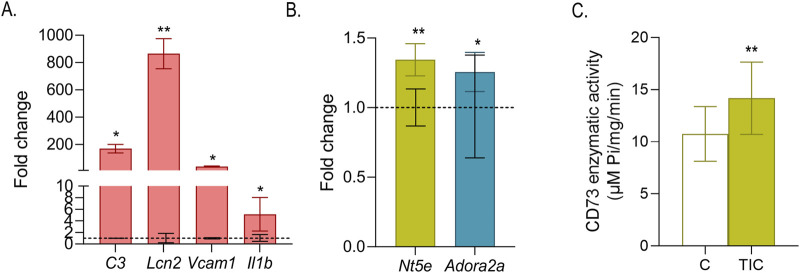
Validation of the TIC-induced neurotoxic reactive astrocyte (nRA) substate. **(A)** qRT-PCR analysis of nRA substate marker genes *C3, Vcam1*, *Lcn2*, and *Il1b* in primary astrocyte cultures stimulated with TIC for 24 h. **(B)** qRT-PCR analysis of *Nt5e* and *Adora2a* expression in astrocyte cultures following 24 h of TIC stimulation. Bars represent fold change (±SEM) relative to control cultures (dashed line), from four different culture, each sample run in duplicate. **(C)** CD73 5′-phosphohydrolase activity measured in control and astrocyte cultures stimulated with TIC for 24 h. Bars represent mean activity (µmol Pi•mg^-1^ protein•min^-1^) ± SEM, from four different culture preparations, with each measurement performed in quadruplicate. Statistical significance is indicated within the graphs (*p < 0.05, **p < 0.01).

Primary astrocytes were stimulated with a combination of human recombinant cytokines (TIC), and acquisition of a reactive phenotype was assessed by profiling the expression of signature nRA marker genes over an 8–32 h time course. Expression of *C3*, *Vcam1*, *Lcn2* and *Il1b* was robustly induced as early as 8 h after TIC stimulation, consistent with the emergence of pro-inflammatory, immune-interactive, and neurotoxic astrocyte phenotype, with each gene displaying a distinct temporal activation profile (Supplementary Figure S1A–D). At 24 h, TIC-stimulated astrocytes exhibited a pronounced inflammatory response (Figure 1A), characterized by marked upregulation of *C3* (169.76 ± 12.4-fold, p < 0.05), *Lcn2* (865.13 ± 110.09, p < 0.01), *Vcam1* (38.64 ± 4.16-fold, p < 0.05), and *Il1b* (5.13 ± 2.91, p < 0.05) relative to non-stimulated cultures. Based on this temporal analysis, in subsequent experiments cultures were stimulated with TIC for 24 h.

To determine whether TIC-induced nRA substate is accompanied by enhanced purinergic signaling, expression of *Nt5e* and *Adora2a* was analyzed across the same 8–32 h time window (Supplementary Figure S1E–F). *Nt5e* expression significantly increased between 8 and 24 h compared with control cultures (Supplementary Figure S1E), whereas *Adora2a* exhibited transient increase between 16 and 24 h (Supplementary Figure S1F). At 24 h, *Nt5e* expression increased to 1.34 ± 0.11-fold (*p* < 0.01), while *Adora2a* expression was modestly but significantly elevated to 1.26 ± 0.14 -fold (*p* < 0.05) relative to control (Figure 1B). Consistent with the transcriptional *Nt5e* upregulation, 5′-phosphohydrolase activity catalyzed by CD73 was enhanced between 24 and 32 h following TIC stimulation. At 24 h time-point, CD73 activity reached 14.2 ± 3.5 µmol Pi•mg^-1^ protein•min^-1^ (*p* < 0.01), which was significantly higher than control (10.8 ± 0.3 µmol Pi•mg^-1^ protein•min^-1^; Figure 1C).

### 3.2 Cellular effects of dual blockade

**FIGURE 2:**
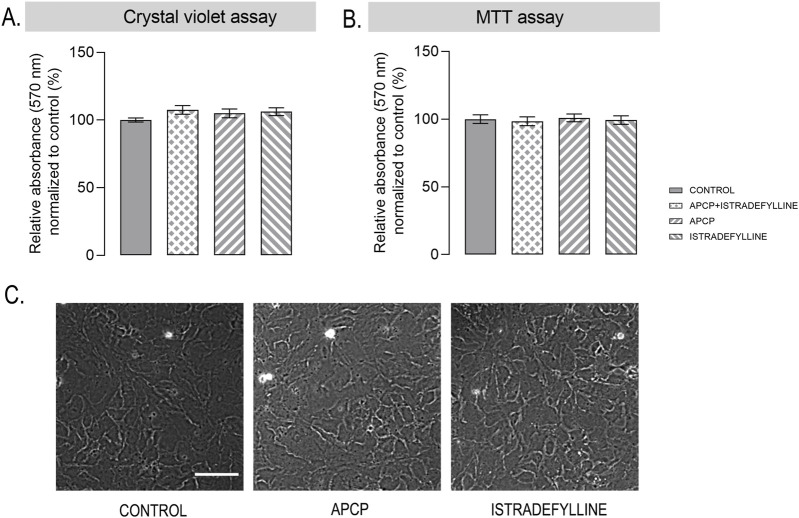
Effects of APCP and istradefylline on astrocyte viability and metabolic activity **(A)** Crystal violet assay and **(B)** MTT assay performed in primary astrocyte cultures treated for 24 h with 100 µM APCP, 10 µM istradefylline, or their combination. Bars in A-B represent mean relative A_570_ ± SEM, relative to control cultures (set to 100%), from three independent astrocyte culture preparations per experimental group, with each measurement performed in triplicate. **(C)** Representative bright-field micrographs of control astrocyte cultures and cultures treated with 100 µM APCP or 10 µM istradefylline. Scale bar = 50 µm.

Given the essential role of adenosine in cellular energy metabolism, before evaluating the efficacy of dual CD73/A_2A_R blockade, potential cellular effects of small molecule inhibitors, APCP and istradefylline, were assessed. Astrocyte viability was examined using crystal violet (CV) and MTT assays in the presence of 100 μM APCP and 10 μM istradefylline. Unchanged CV absorbance indicated that the inhibitors, either alone or combined, did not affect the total number of adherent viable cells (Figure 2A), nor did they impair mitochondrial metabolic activity as assessed by MTT reduction (Figure 2B).

Consistently, bright-field micrographs revealed that astrocytes treated with APCP or istradefylline preserved usual morphology, an intact monolayer, showing no evidence of cell shrinkage, process retraction, or swelling (Figure 2C). Together, these findings indicate that the inhibitors in applied concentrations were well tolerated and did not induce cytotoxic or stress-related effects.

### 3.3 Cell shape and cytoskeletal re-organization following dual blockade

**FIGURE 3:**
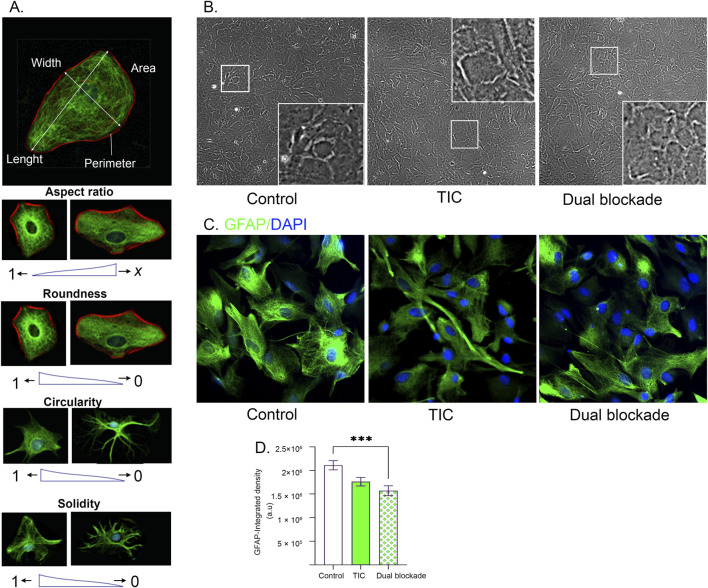
Astrocyte shape and cytoskeletal organization following dual CD73/A_2A_R blockade. **(A)** Schematic examples illustrating how individual shape descriptors reflect specific aspects of astrocyte morphology and structural remodeling. **(B)** Representative bright-field micrographs of astrocyte cultures across experimental groups (magnification ×10), used for morphometric analysis and shape descriptor quantification in *ImageJ*. In each micrograph, a selected region is digitally enlarged (×20) to highlight morphological details. **(C)** GFAP-label immunofluorescence illustrating changes in astrocyte morphology in TIC-stimulated cultures and cultures treated with dual blockade relative to control. **(D)** Quantitative analysis of GFAP fluorescence intensity across experimental groups. Bars represent mean GFAP integrated density (a.u.) ± SEM measured across 10 random non-overlapping fields from three independent astrocyte culture preparations per experimental group.

As morphological remodeling represents an early indicator of astrocyte activation, the effects of dual CD73/A_2A_R blockade were further evaluated by assessing changes in astrocyte morphology using dual GFAP/DAPI-labeled confocal images. In contrast to flat, polygonal shape of control astrocytes, TIC stimulation induced a hypertrophy characterized by elongation and the emergence of cellular processes. Astrocytes subjected to dual blockade mostly normalized cell body, while many retained prominent cellular processes (Figure 3A). While GFAP immunofluorescence did not reveal apparent alterations in intermediate filament organization between control, TIC-stimulated cultures and those treated with dual blockade, GFAP signal intensity was significantly lower in cultures subjected to dual blockade relative to control cultures (Figure 3B).

These observations were further validated by quantitative morphometric analysis, which included assessment of shape descriptors - cell area, perimeter, aspect ratio, circularity, and solidity. Representative examples illustrating how each descriptor reflects specific aspects of astrocyte morphology and structural remodelling are shown in Figure 3C, while representative bright-field images of control cultures, TIC-stimulated cultures, and cultures treated with dual blockade are shown in Figure 3D.

In TIC-stimulated cultures, astrocytes exhibited increased cell surface area, perimeter, aspect ratio, and solidity, together with reduced circularity compared with control cultures, consistent with a hypertrophic and anisotropic phenotype. Following dual CD73/A_2A_R blockade, cell surface area and perimeter were reduced to near-control values, whereas aspect ratio, circularity, and solidity remained unchanged. This pattern is consistent with attenuation of astrocyte hypertrophy and preserved anisotropy, contour irregularity, and structural compactness. Numerical values for shape descriptors and statistical comparison between experimental groups are shown in Table 3.

**TABLE 3:** Morphometric analysis.

Treatment	C	TIC	Dual blockade
Shape descriptors			
Area (μm^2^)	1,698 ± 65	2,837 ± 119^*^	2,168 ± 102^*#^
Perimeter (μm)	162.5 ± 3.5	223.1 ± 6.1^*^	188.3 ± 4.96^*#^
Aspect ratio	1.54 ± 0.03	1.73 ± 0.05^*^	1.66 ± 0.06
Roundness	0.68 ± 0.01	0.62 ± 0.02^*^	0.66 ± 0.02
Solidity	0.95 ± 0.01	0.92 ± 0.08^*^	0.94 ± 0.01

Mean area (μm^2^), perimeter (μm) and shape descriptor values expressed as a mean ± SEM, from n ≥ 100 cells in 3 random and non-overlapping microscopic fields.

*p < 0.05 vs. control; #*p* < 0.05 vs. TIC.

### 3.4 Modulation of CD73 and A_2A_R by dual blockade

**FIGURE 4:**
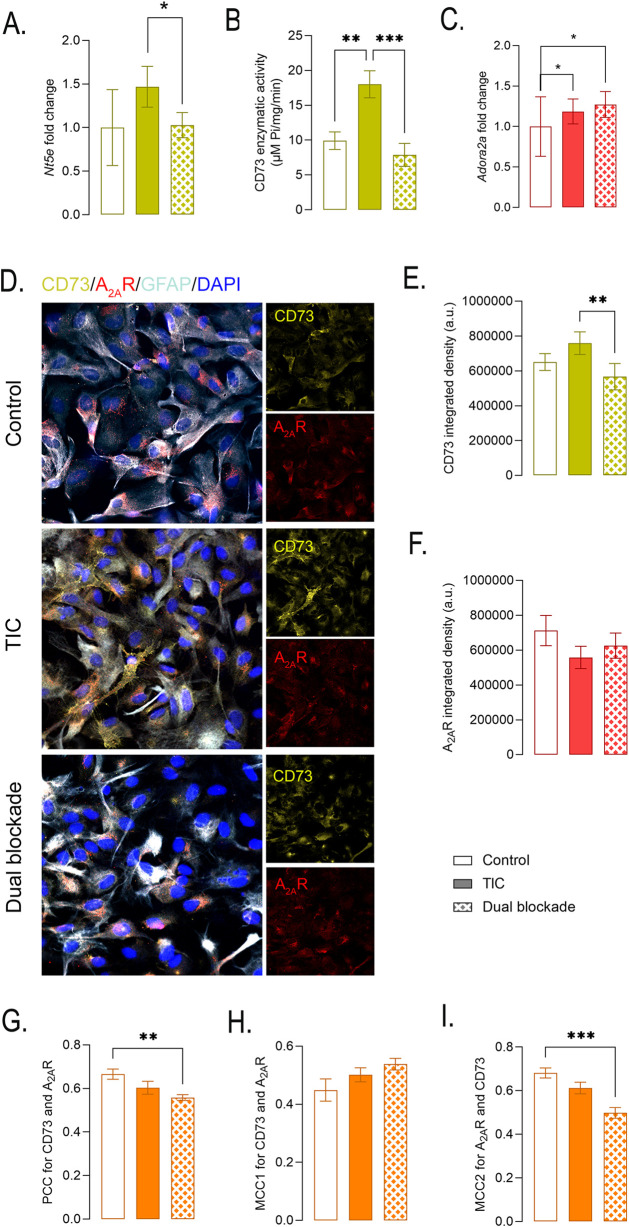
Effects of dual CD73/A2AR blockade on expression and spatial organization of the molecular targets CD73 and A2AR. **(A)** *Nt5e* gene expression, **(B)** CD73 5′-phosphohydrolase activity (µmol Pi•mg^-1^ protein•min^-1^), and **(C)** *Adora2a* gene expression across experimental groups. Bars represent mean ± SEM from n ≥ 5 independent measurements, derived from at least three independent astrocyte culture preparations. **(D)** Representative multi-label confocal images showing CD73 (*yellow*), A2AR (*red*), GFAP (*green*), and nuclei (DAPI, *blue*). Images were acquired from 10 random non-overlapping fields per experimental group across three independent astrocyte culture preparations. **(E,F)** Quantitative analysis of CD73 and A_2A_R fluorescence intensity. **(G)** Pearson’s correlation coefficient (PCC) across experimental groups. **(H,I)** Manders’ correlation coefficients, **(H)** MCC1 for CD73 overlapping with A2AR and **(I)** MCC2, for A2AR overlapping with CD73, determined from confocal image analysis. Statistical significance indicated within the graphs: *p < 0.05; **p < 0.01; ***p < 0.001.

To assess whether dual CD73/A_2A_R blockade modulates the expression of its own molecular targets under inflammatory conditions, *Nt5e* and *Adora2a* expression was analyzed. Dual blockade significantly attenuated TIC-induced upregulation of *Nt5e* (Figure 4A), reducing its expression from 1.47 ± 0.23-fold in TIC-stimulated cultures to near-control levels (1.02 ± 0.14; p < 0.05). Consistent with this transcriptional normalization, dual blockade also reduced the 5′-phosphohydrolase activity of CD73 (7.89 ± 1.64 µmol Pi•mg^-1^ protein•min^-1^; p < 0.001) compared with TIC-stimulated astrocytes (Figure 4B). Notably, neither APCP nor istradefylline administered alone induced comparable effects on *Nt5e* expression (Supplementary Figure S1H), indicating that coordinated inhibition of CD73 enzymatic activity and A_2A_R signaling is required to normalize *Nt5e* expression under inflammatory conditions. Dual blockade did not significantly alter expression of *Adora2a* (Figure 4C).

The differential effects of dual CD73/A_2A_R blockade on CD73 and A_2A_R were further confirmed by double-label confocal immunofluorescence analysis (Figure 4D). A significant reduction in CD73 fluorescence intensity was observed in cultures treated with dual blockade compared with TIC (Figure 4E), whereas A_2A_R fluorescence intensity was not significantly altered (Figure 4F). The PCC value progressively decreased from control (0.66 ± 0.02) to TIC (0.60 ± 0.03) and cultures treated with dual blockade (0.56 ± 0.01; *p* < 0.01), indicating reduced signal intensity correlation following dual blockade (Figure 4G). In contrast, determination of MCC, which assess pixel co-occurrence of two signals (co-localization), revealed no significant changes in MCC1 (CD73 overlapping with A_2A_R) (Figure 4H), whereas, it showed significant reduction in MCC2 (A_2A_R overlapping with CD73) in cultures subjected to dual blockade compared with TIC (Figure 4I, *p* = 0.01). These changes indicate a selective reduction in spatial association of A_2A_R with CD73-enriched membrane regions following dual blockade.

### 3.5 Effects of dual CD73/A_2A_R blockade on oxidative stress and antioxidant capacity

**FIGURE 5:**
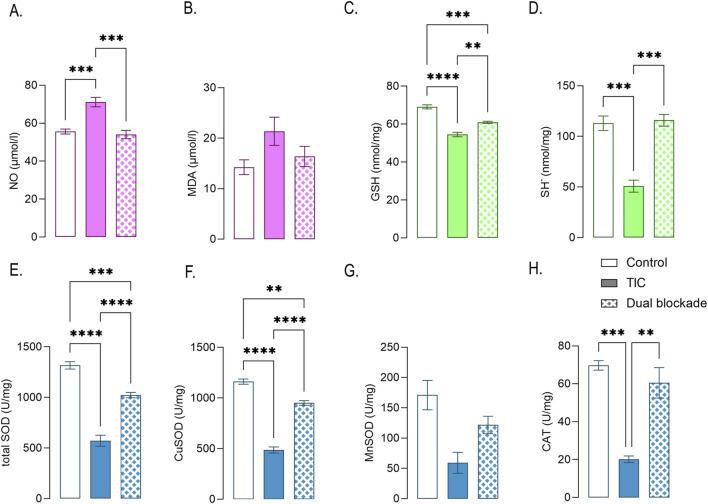
Effects of dual CD73/A2AR blockade on oxidative stress and antioxidant defenses in TIC-induced reactive astrocytes. Pro-oxidant markers measured in culture medium: **(A)** Nitric oxide (NO) and **(B)** malonaldehyde (MDA). Non-enzymatic antioxidant parameters measured in cell lysates: **(C)** Glutathione (GSH) and **(D)** total sulfhydryl groups (SH^−^). Enzymatic antioxidant activities measured in cell lysates: **(E)** total SOD, **(F)** Cu/Zn-SOD, **(G)** Mn-SOD, and **(H)** CAT. Bars represent mean ± SEM from 3-5 independent astrocyte culture preparations. Statistical significance is indicated within the graphs (*p < 0.01, **p < 0.001).

The effects of dual CD73/A_2A_R blockade on oxidative stress and antioxidant capacity are shown in Figure 5. Nitric oxide (NO) levels were significantly increased in TIC cultures (71.14 ± 2.48 μmol/L) compared with control (55.59 ± 1.32 μmol/L; p < 0.001) and were reduced to control values in cultures treated with dual blockade (54.05 ± 2.21 μmol/L; p < 0.001 in respect to TIC) (Figure 5A). Malondialdehyde (MDA) levels were elevated in TIC cultures (21.37 ± 2.79 μmol/L) relative to control (14.25 ± 1.46 μmol/L) and were reduced following dual blockade (16.39 ± 2.00 nmol/mg), although this change did not reach statistical significance (Figure 5B).

Dual blockade restored intracellular redox homeostasis. Glutathione (GSH) levels, reduced in TIC astrocytes (54.48 ± 1.18 nmol/mg; p < 0.0001) compared with control (69.09 ± 1.06 nmol/mg), were significantly increased in cultures treated with dual blockade (60.93 ± 0.59 nM/mL; p < 0.01 in respect to TIC) (Figure 5C). Likewise, total sulfhydryl (SH^−^) content, decreased in TIC cultures (50.88 ± 5.96 nmol/mg; p < 0.0001) relative to control (113.1 ± 7.14 nmol/mg), was restored to near-control levels following dual blockade (116.0 ± 5.79 nmol/mg; p < 0.01 in respect to TIC) (Figure 5D).

Dual CD73/A_2A_R blockade also preserved enzymatic antioxidant defenses. Total SOD activity was reduced in TIC cultures (572.4 ± 54.29 U/mg; p < 0.0001) and markedly increased in following dual blockade (1,022 ± 26.71 U/mg; p < 0.0001 in respect to TIC) (Figure 5E), mainly due to changes in cytosolic Cu/Zn-SOD (TIC: 486.6 ± 30.21 U/mg; p < 0.0001; dual blockade: 951.3 ± 23.54 U/mg; p < 0.0001 in respect to TIC) (Figure 5F). MnSOD showed a similar trend, with reduced activity in TIC cultures (59.25 ± 17.25 U/mg) compared with control (171.1 ± 24.3 U/mg, p < 0.05) and partial recovery following dual blockade (121.8 ± 14.42 U/mg), although without statistical significance (Figure 5G). Catalase (CAT) activity, decreased in TIC cultures (20.17 ± 1.75 U/mg; p < 0.001) relative to control (69.72 ± 2.52 U/mg), was restored to near-control levels in following dual blockade (60.51 ± 8.04 U/mg; p < 0.01 in respect to TIC) (Figure 5H).

### 3.6 Regulation of inflammatory signaling and astrocyte–microglia communication by dual blockade

The observed attenuation of oxidative stress, restoration of redox balance and attenuation of astrocyte hypertrophy strongly suggest that dual CD73/A_2A_R blockade has the capacity to modulate inflammatory signaling in reactive astrocytes. Therefore, key inflammatory mediators were further assessed at both the transcriptional and protein levels. As summarized in Table 4, TIC stimulation induced a broad inflammatory transcriptional program typical of the nRA substate, whereas dual blockade selectively modulated distinct components of the inflammatory network.

**TABLE 4:** Quantitative gene expression analysis of inflammatory genes in control culture, TIC culture and culture treated with dual blockade.

Target gene	Control	TIC	Dual blockade
*Nt5e*	1.00 ± 0.46	1.47 ± 0.23^a^	1.03 ± 0.14^b^
*Adora2a*	1.00 ± 0.37	1.43 ± 0.19^a^	1.46 ± 0.17^a^
*C3*	1.00 ± 0.65	65.95 ± 9.64^a^	45.84 ± 7.17^a^ ^,^ ^b^
*Vcam1*	1.00 ± 0.22	20.12 ± 2.61^a^	10.18 ± 1.38^a^ ^,^ ^b^
*Lcn2*	1.00 ± 0.21	13.69 ± 1.98^a^	12.3 ± 2.06^a^
*Il10*	1.00 ± 0.40	1.38 ± 0.35	1.07 ± 0.41
*Il1b*	1.00 ± 0.26	3.34 ± 0.78^a^	4.87 ± 0.43
*Il6*	1.00 ± 0.22	21.88 ± 3.37^a^	33.24 ± 4.23^a^
*Tnf*	1.00 ± 0.61	2.48 ± 1.18^a^	5.96 ± 3.16^a^
*Ccl2*	1.00 ± 0.29	40.54 ± 5.64^a^	25.44 ± 3.60^a^
*Cxcl16*	1.00 ± 0.21	19.67 ± 2.34^a^	20.44 ± 2.13^a^
*Rela*	1.00 ± 0.01	2.81 ± 0.33^a^	2.47 ± 0.39^a^
*Stat3*	1.00 ± 0.24	2.20 ± 0.36^a^	2.24 ± 0.38^a^
*Cebpb*	1.00 ± 0.79	0.78 ± 0.68	0.54 ± 0.41
*Nfe2l2*	1.00 ± 0.28	3.11 ± 0.21^a^	3.83 ± 0.46^a^
*Prkacb*	1.00 ± 0.22	1.30 ± 0.12	1.33 ± 0.14
*Nos2*	1.00 ± 0.86	22.59 ± 5.41^a^	13.90 ± 2.99^a^ ^,^ ^b^
*Lif*	1.00 ± 0.04	4.61 ± 0.43^a^	10.16 ± 0.99^a^ ^,^ ^b^
*Serping1*	1.00 ± 0.13	5.51 ± 0.52^a^	4.39 ± 0.42^a^
*S100a10*	1.00 ± 0.01	0.65 ± 0.04^a^	0.62 ± 0.02^a^
*Apoe*	1.00 ± 0.06	1.20 ± 0.10	1.22 ± 0.12
*Gfap*	1.00 ± 0.17	0.69 ± 0.09	0.54 ± 0.07^a^ ^,^ ^b^

^a^ Denotes statistical significance at *p* < 0,05 in respect to control.

^b^ Denotes statistical significance at *p* < 0.05 in respect to TIC.

**FIGURE 6:**
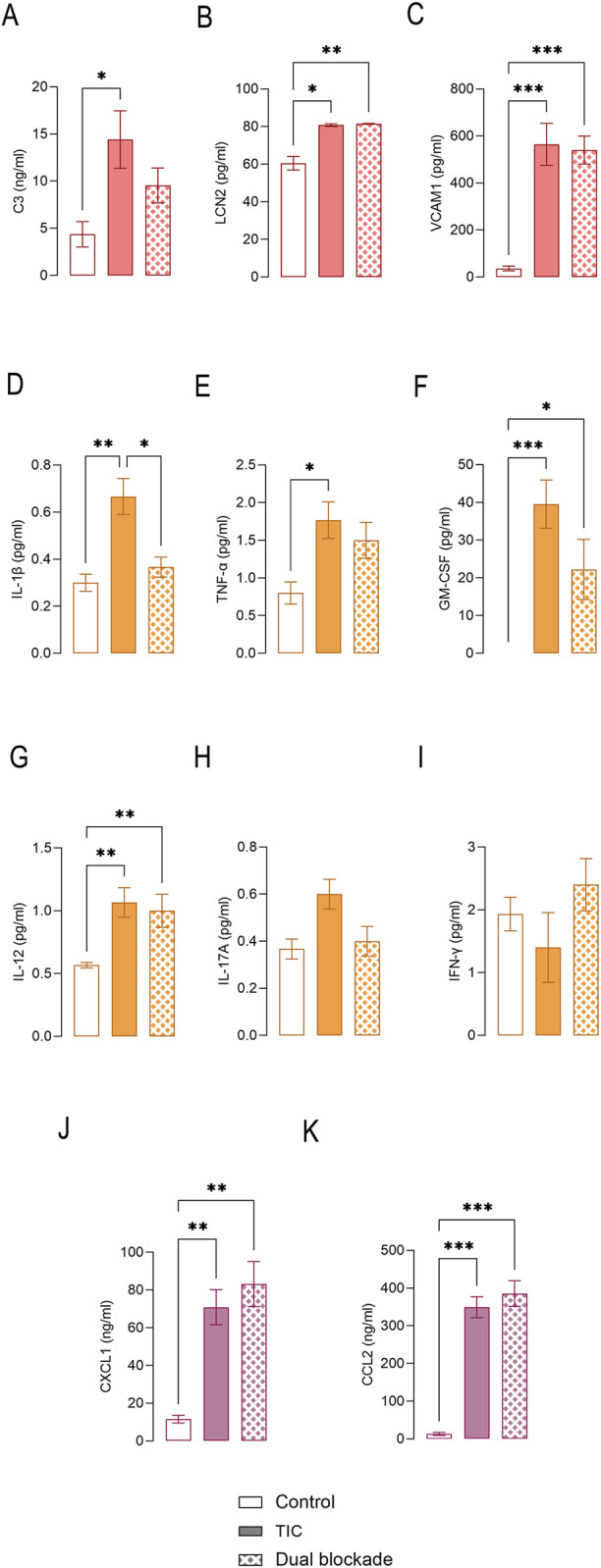
Effects of dual CD73/A_2A_R blockade on secretion of inflammatory mediators in TIC-induced reactive astrocytes. Concentrations of secreted proteins were determined by ELISA in culture supernatants. Shown are levels of **(A)** Complement component 3 C3; **(B)** Lipocalin-2 (LCN2); **(C)** Vascular cell adhesion molecule-1 (VCAM1); **(D)** Interleukin-1 β (IL-1β); **(E)** Tumor necrosis factor–α (TNFα); **(F)** Granulocyte-Macrophage Colony-Stimulating Factor (GM-CSF); **(G)** Interleukin-12 (IL-12); **(H)** Interleukin-17 (IL-17A); **(I)** Interferon- γ (IFNγ); **(J)** CXCL1, and **(K)** CXCL16. Bars represent mean cytokine concentration (as indicated) ± SEM based on three independent culture pre parations for each experimental group, with each sample analyzed in duplicate.

At the transcriptional level, dual blockade significantly reduced the expression of *C3* and *Vcam1*, whereas *Lcn2* expression remained elevated relative to TIC-treated cultures. Under the tested conditions, this modulatory effect was not recapitulated by either inhibitor alone (Supplementary Figure S2). This pattern was mirrored at the protein level, with reduced secretion of C3 and VCAM1 but persistently elevated LCN2 in cultures treated with dual blockade (Figures 6A–C). Among pro-inflammatory cytokines, dual blockade also selectively suppressed TIC-induced gene expression, although the transcriptional effects were not consistently reflected at the protein level. Specifically, levels of IL-1β, GM-CSF, and IL-17A were normalized in cultures treated with dual blockade (Figures 6D,F,H), whereas TNF-α and IL-12 levels remained elevated (Figures 6E,G). Neither TIC stimulation nor dual blockade significantly affected IFN-γ levels (Figure 6I). Dual blockade did not affect chemokine- driven astrocyte-microglia/immune cell communication, since TIC- induced induction of Ccl2 and Cxcl16 were paralleled by elevated CCL2 and CXCL1 protein levels (Figures 6J,K).

Within the group of inflammation-associated effector and regulatory genes, dual blockade attenuated TIC-induced *Nos2* expression, consistent with reduced NO production. Expression of *Lif* coding Leukemia inhibitory factor (LIF), which supports astrocytes to survive oxidative stress and *Serping1*, which encodes C1-inhibitor primarily serving as a regulator of the complement systems, remained significantly elevated following dual blockade. Genes associated with astrocyte structural remodeling (*Gfap* and *S100a10*) were downregulated by TIC and remained suppressed following dual blockade. Finally, expression of major inflammatory transcriptional regulators *Rela* (Nfkb) and *Stat3* remained elevated after dual blockade, whereas the antioxidant regulator *Nfe2l2* was robustly induced under both conditions, consistent with re-established redox-protective responses.

### 3.7 Assessing the neurotoxic potential of the TIC-stimulated astrocyte secretome following dual blockade

**FIGURE 7:**
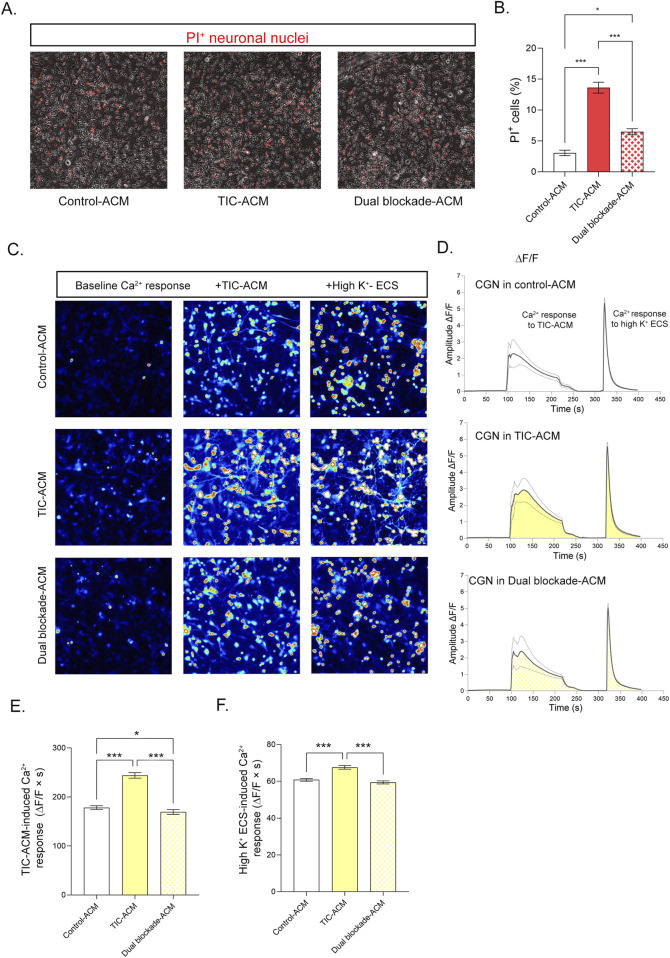
Effects of dual CD73/A2AR blockade on the neurotoxic potential of TIC-induced reactive astrocyte secretome. **(A)** Representative bright-field micrographs of cerebellar granule neuron (CGN) cultures maintained in respective ACMs (control-ACM, TIC-ACM, dual blockade-ACM) for 24 h, overlaid with propidium iodide (PI) staining. **(B)** Quantitative analysis of PI-positive nuclei in CGNs. Bars represent the mean number of PI^+^ neuronal nuclei (%, ± SEM) from 10 non-overlapping fields per experimental group across two independent neuronal culture preparations, maintained in ACM derived from four TIC-stimulated astrocyte cultures. **(C)** Representative images of Fluo-4 -loaded CGNs maintained for 24 h in ACMs (control-ACM, TIC-ACM, or dual blockade-ACM), showing baseline intracellular Ca^2+^ response and the responses following acute exposure to TIC-ACM and high K^+^ ECS. **(D)** Representative time-lapse traces of intracellular Ca^2+^ dynamics recorded over 400 s, including baseline perfusion with ECS (0–90 s), response to addition of TIC-ACM (100–220 s), and response to high K^+^ ECS added at 320 s. **(E)** Quantification of the TIC-ACM -induced Ca^2+^ response integrals presented in **(D)**. **(F)** Quantification of the K^+^-induced Ca^2+^ response integral. Bars in E-F represent the mean Ca^2+^ response integral (± SEM; ΔF/F × s) from two independent neuronal cultures treated with TIC-ACMs derived from four astrocyte cultures. Statistical significance within the graphs: *p < 0.01; **p < 0.001; ***p < 0.0001.

To evaluate the ability of dual blockade to attenuate the neurotoxic potential of the nRA secretome, neuronal cell death and intracellular Ca^2+^ dynamics were quantified in cerebellar granule neurons (CGNs) maintained for 24 h in astrocyte-conditioned media (ACM) obtained from control (control-ACM), TIC-stimulated (TIC-ACM), or dual blockade–treated astrocyte cultures (dual blockade-ACM) (Figure 7). Neuronal death was assessed using propidium iodide (PI) staining, and representative micrographs are shown in Figure 7A. CGN cultures maintained in TIC-ACM exhibited an almost threefold higher number of PI-positive neurons compared to those maintained in control-ACM (p < 0.001), confirming a pronounced neurotoxic effect of nRA-derived factors. In contrast, CGNs maintained for 24 h in dual blockade-ACM displayed approximately 40% fewer PI-positive cells compared to TIC-ACM-treated cultures (p < 0.001), although neuronal death remained elevated relative to control-ACM (p < 0.01) (Figure 7B).

Intracellular Ca^2+^ dynamics were further analyzed as readout of neuronal homeostasis and excitability. Figure 7C shows representative micrographs of CGNs maintained for 24 h in the respective ACMs, including baseline Ca^2+^ responses and responses to additional brief exposure to TIC-ACM and to high-K^+^ ECS solution. Mean fluorescence traces recorded over 120 s following addition of TIC-ACM or high-K^+^ ECS are presented in Figure 7D. A significantly increased intracellular Ca^2+^ response was observed in CGNs maintained in TIC-ACM (244.0 ± 5.64; p < 0.0001) compared to control-ACM (178.2 ± 3.83), indicating pronounced Ca^2+^ dysregulation (Figure 7E). In contrast, CGNs maintained in dual blockade-ACM exhibited a significantly lower Ca^2+^ response in respect to TIC-ACM (169.4 ± 4.82; p < 0.0001), which was comparable to control-ACM levels (p < 0.01).

To assess neuronal excitability, CGNs pre-incubated in the respective ACMs were subsequently challenged with depolarizing high-K^+^ ECS (Figures 7D,F). Robust Ca^2+^ responses were elicited in all groups (control-ACM: 60.82 ± 0.74; TIC-ACM: 67.59 ± 1.00; dual blockade-ACM: 59.41 ± 0.86). Although minor but statistically significant differences were detected between groups (p < 0.001), the overall magnitude of depolarization-induced Ca^2+^ influx remained comparable, indicating preserved neuronal excitability following exposure to the respective ACMs.

### 3.8 Bioinformatics network analysis

**FIGURE 8:**
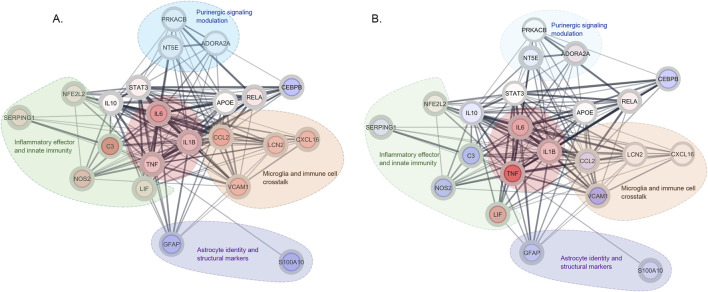
Network analysis of differentially expressed genes and their predicted functional associations. Protein-protein interactions networks show significantly altered genes and their predicted functional associations (edges) in TIC-stimulated astrocytes relative to control **(A)** and in TIC-stimulated astrocytes treated with dual CD73/A_2A_R blockade **(B)**. Each node represents a gene that is significantly upregulated (*red*) or downregulated (*blue*). Thicker and darker edges indicate higher confidence in the predicted interactions and functional associations between gene products. Dashed outlines denote manually annotated functional modules based on known biological roles.

The inflammatory gene expression was used to compare the global organization of inflammatory signaling in TIC-stimulated cultures and cultures treated with dual blockade. In TIC-stimulated cultures, analyzed genes formed an interconnected network dominated by a core inflammatory cytokines module (TNFα, IL1-β, IL6). The core was strongly connected with downstream effectors, resolved into four functional modules: the inflammatory effector/innate immune module (NOS2, C3, Lif, SERPING1), chemokine-mediated astrocyte-microglia/immune cells communication module (VCAM1, CXCL16, CCl2), purinergic signaling/modulation module (CD73, A_2A_R, PKC), and astrocyte identity/structural module (GFAP, S100A10) (Figure 8A).

Following dual blockade, while the overall modular organization of the network was preserved (Figure 8B), marked changes appeared in internal connectivity. The core inflammatory signaling module remained prominent, with TNFα, IL1-β, IL6 maintaining the central positions, whereas connectivity within the inflammatory effector/innate immune module was reduced, particularly for nodes associated with NOS2, C3, and VCAM1. The chemokine-mediated microglia/immune cells communication module remained largely unaffected following dual blockade, with CXCL16 and CCL2 maintaining strong network integration. The purinergic modulation module (*NT5E*, *ADORA2A*, PRKACB) remained connected to both upstream regulators and downstream effectors, while the astrocyte identity/structural module (GFAP and S100A10) displayed similar positioning in TIC networks and networks in cultures treated with dual blockade.

## 4 Discussion

It is well established that the extracellular ATP/adenosine ratio critically determines inflammatory tone in the CNS (Aminin and Illes, 2021). Whereas inhibition of CD39 primarily interferes with ATP-to-AMP conversion, and broadly alters both P2 and P1 receptor signaling, selective targeting of CD73 enables interrogation of adenosine-dependent downstream pathways mediated through P1 receptors. In the present study, we focused on a defined enzyme-receptor coupling module to examine whether modulation of the CD73/A_2A_R axis can attenuate the neurotoxic reactive astrocyte (nRA) substate induced by the combination of microglia-derived TNFα, IL-1α, and C1q (TIC). This cytokine cocktail activates a well-established inflammatory cascade in astrocytes, in which TNFα and IL-1α converge on the upstream kinase TAK1 (Soto-Díaz et al., 2020; Yang et al., 2010), leading to activation of NF-κB and MAPK signaling and initiation of early pro-inflammatory gene transcription (Soto-Díaz et al., 2020; Hua et al., 2002). Downstream amplification through C/EBPβ, IL-6 production (Cardinaux et al., 2000), and JAK/STAT3 signaling stabilizes the reactive phenotype, while C1q provides a complementary signal that enables full polarization toward a neurotoxic astrocyte substate by engaging complement-associated effector programs (Liddelow et al., 2017; Matusova et al., 2023).

Consistent with this framework, TIC stimulation in our model recapitulated the transcriptional and functional hallmarks of a highly reactive, immune-competent nRA phenotype. TIC stimulation elicited morphological hallmarks of astrocyte reactivity, such as process hypertrophy and structural remodeling, without GFAP upregulation, as previosly described in this paradigm (Zimmermann, 2000). TIC robustly induced the core inflammatory cytokines IL-6, IL-1β, and TNFα, which constitute a self-sustaining inflammatory loop driving neuroinflammation (Mallick et al., 2025). In parallel, marked upregulation of C3, VCAM1, and LCN2 was observed, reflecting activation of effector programs that stabilize a pro-inflammatory, neurotoxic, and immune-interactive astrocyte state (Tan et al., 2024; Peoples et al., 2021; Gimenez et al., 2004). This inflammatory profile was accompanied by induction of IL-17, IL-12, and GM-CSF, indicating engagement of Th17-, Th1-, and myeloid-associated immune pathways (Kang et al., 2010). Moreover, TIC provoked pronounced oxidative stress, as evidenced by elevated NO production, lipid peroxidation, impaired antioxidant defences, and robust induction of chemokines that facilitate astrocyte–microglia and astrocyte–immune cell communication.

Importantly, our data identify recruitment of the CD73/A_2A_R signaling axis as an integral part of the TIC-induced nRA substate. CD73 was engaged early following TIC stimulation, with *Nt5e* transcriptional upregulation preceding increased membrane expression and enzymatic activity, consistent with predominant transcriptional regulation (Resta et al., 1993). This pattern aligns with established CREB-dependent control of *Nt5e* transcription (Spychala et al., 1999), together with additional regulation by SP1, AP-2, and SMAD family members that confer astrocyte-specific and context-dependent expression (Resta et al., 1993). Accordingly, early *Nt5e* induction likely reflects cAMP-CREB and IL-1β signaling combined with MAPK/AP-1 activation, whereas sustained expression at later stages may result from prolonged NF-κB activity and secondary IL-6/STAT3 signaling that stabilizes the neurotoxic astrocyte phenotype (Adzic et al., 2024; Kordaß et al., 2018).

In contrast, *Adora2a* exhibited only transient transcriptional upregulation in TIC-stimulated cultures without a corresponding increase in A_2A_R membrane protein levels, consistent with complex post-transcriptional regulation of receptor expression (Chu et al., 1996; Lee et al., 2003). Nevertheless, TIC enhanced spatial coupling of CD73 and A_2A_R within same membrane microdomains, a configuration that facilitates localized adenosine delivery (Augusto et al., 2021) and amplifies A_2A_R signaling without requiring increased receptor abundance. While this effect was not directly demonstrated, the spatial organization likely strengthens cAMP-dependent signaling during early astrocyte remodeling.

A central question addressed in this study was whether pharmacological dual blockade of CD73 and A_2A_R can constrain polarization toward the nRA substate. Dual blockade inhibited TIC-induced CD73 enzymatic activity and altered spatial proximity between CD73 and A_2A_R, likely attenuating cAMP-CREB- and PKA-dependent effector programs associated with neurotoxic astrocyte activation (Launay et al., 2025; Augusto et al., 2021; Matyash et al., 2017; Simões et al., 2022). Importantly, dual blockade also normalized expression of its primary molecular target, CD73, suggesting that its effects extend beyond acute signal inhibition and may confer more durable reprogramming of astrocyte signaling capacity.

At the morphological level, dual blockade selectively attenuated hypertrophic features of astrocyte activation without inducing full reversion to a resting phenotype. Parameters dependent on rapid cytoskeletal dynamics (Schiweck et al., 2018) were normalized, whereas features requiring transcription-dependent structural remodeling remained largely unchanged (Herrmann et al., 2008). Consistent with this, GFAP expression and intermediate filament organization were not restored, indicating that dual blockade acts upstream of transcriptional programs governing structural gliosis (Sofroniew and Vinters, 2010; Sofroniew, 2020).

Gene and protein expression analyses further revealed that CD73/A_2A_R blockade exerts a highly selective modulatory effect on the inflammatory network that was not recapitulated by either inhibitor alone under the tested conditions. Dual blockade normalized IL-1β secretion while leaving IL-6 and TNFα levels largely unchanged, indicating preservation of core inflammatory signaling. A similar selective pattern was observed for other inflammatory mediators. Namely, key nRA markers C3 and VCAM1 were markedly reduced, whereas the pan-reactive marker LCN2 remained elevated. GM-CSF and IL-17A secretion was attenuated, suggesting overall weakening of the nRA profile (Hu et al., 2017; Shiomi et al., 2016; Gajtkó et al., 2020), while IL-12 levels were preserved. Sustained expression of chemokines such as CXCL1, CXCL16, and CCL2 further indicates that astrocyte-mediated microglia and immune cell recruitment capacity remains intact following dual blockade.

The most pronounced effects of dual blockade were observed at the level of oxidative balance. Suppression of *Nos2* expression and normalization of NO production, together with robust induction of *Nfe2l2* (Nrf2), which is the master upstream regulator of antioxidant genes (Ngo and Duennwald, 2022), resulted in restoration of intracellular antioxidant capacity, a pattern consistent with activation of NRF2-dependent antioxidant programs. Dual blockade also enhanced glutathione recycling, thiol-based redox buffering, and increased activity of key antioxidant enzymes. Collectively, these changes indicate that pharmacological blockade of CD73/A_2A_R induces a shift toward a redox-stable astrocyte substate (Saha et al., 2022; Mayer et al., 2024) and reprograms strong neurotoxic activity of TIC-stimulated astrocytes (Liddelow et al., 2017) toward a less neurotoxic functional phenotype. Indeed, dual blockade markedly attenuated TIC astrocyte-induced neuronal Ca^2+^ dysregulation and significantly reduced cytotoxicity, confirming that reprogramming of the astrocyte redox and inflammatory profile translates into a measurable decrease in neurotoxic secretory activity.

Integration of network analysis with molecular, biochemical, morphological and functional data supports a model in which dual CD73/A_2A_R blockade operates within a defined pharmacological window of modulation. The selective effects of dual blockade are best explained by interference with membrane-proximal cAMP-dependent signaling, which governs cAMP- and redox-sensitive inflammatory effector pathways while leaving cytokine- and chemokine-driven responses largely intact. The lack of changes in major transcriptional regulators, including NF-κB and STAT3, as well as in core inflammatory cytokines and chemokines involved in astrocyte–microglia–immune cell interactions, indicates that dual blockade, at least under the tested experimental conditions, does not suppress the central inflammatory transcriptional machinery. In contrast, normalization of C3, VCAM1, and *Nos2*/NO levels together with restoration of antioxidant capacity points to a shift toward a less neurotoxic astrocyte profile. Such targeted reconfiguration of inflammatory networks may represent potential strategy to limit chronic astrocyte-driven neurotoxicity without compromising essential immune and homeostatic functions (O et al., 2025). It should be noted that the present study relies on pharmacological modulation of the CD73/A_2A_R axis, and although the small-molecule inhibitors employed are widely used experimental tools, potential off-target effects cannot be fully excluded. Accordingly, the current findings should be interpreted as evidence for functional involvement of the CD73/A_2A_R signaling axis rather than definitive genetic causality. Future studies employing genetic knockdown or conditional knockout approaches, as well as validation in disease-relevant models, will be necessary to establish mechanistic specificity and further evaluate therapeutic potential.

## Data Availability

The original contributions presented in the study are included in the article/Supplementary Material, further inquiries can be directed to the corresponding author.
