# Supplementary material for: Dual CD73/A2AR blockade modulates the neurotoxic astrocyte phenotype without disrupting core inflammatory signaling
Source: Front Pharmacol. 2026 Apr 22;17:1778355. doi: 10.3389/fphar.2026.1778355 (PMC13143737; doi:10.3389/fphar.2026.1778355)
Supplement: Supplementary file 1 [file Supplementaryfile1.pdf]

Supplementary Material

1 Supplementary Figures and Tables

1.1 Supplementary Figure 1

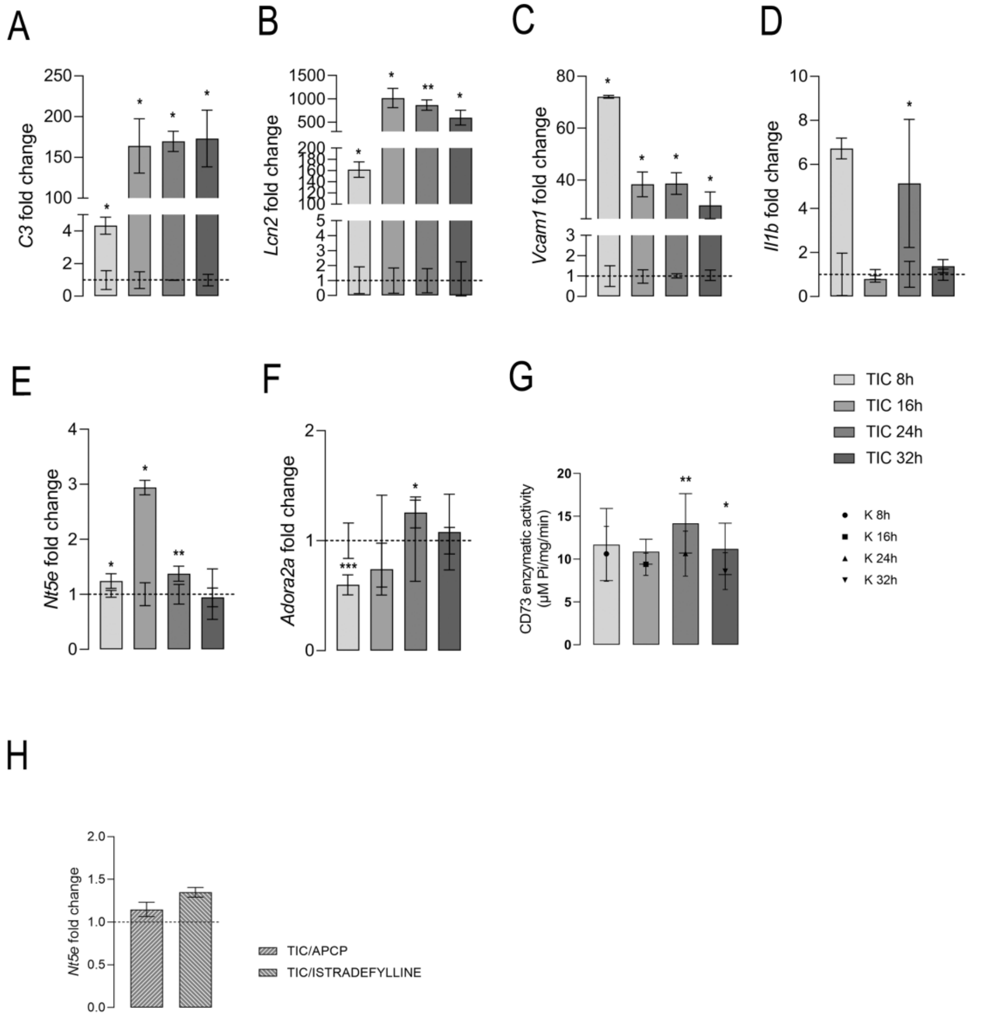

Legend to Supplementary Figure 1.

(A–F) Temporal gene expression profiles of *C3* (A), *Lcn2* (B), *Vcam1* (C), *Il1b* (D), *Nt5e* (E), and *Adora2a* (F) at 8–32 h following TIC stimulation. Bars represent mean fold change ± SEM relative to control cultures (dashed line) for each time point, obtained from three independent astrocyte culture preparations, with each sample analyzed in duplicate.

(G) Temporal profile of CD73 5′-phosphohydrolase activity measured at 8–32 h following TIC stimulation. Bars represent mean enzymatic activity (µmol Pi•mg^{-1} protein•min^{-1} ± SEM) from five independent astrocyte culture preparations, each analyzed in duplicate.

(H) Effects of APCP or istradefylline administered individually on *Nt5e* expression in astrocyte cultures stimulated with TIC for 24 h.

Statistical significance is indicated within the graphs (**p* < 0.05; ***p* < 0.01).

1.2. Supplementary Figure 2

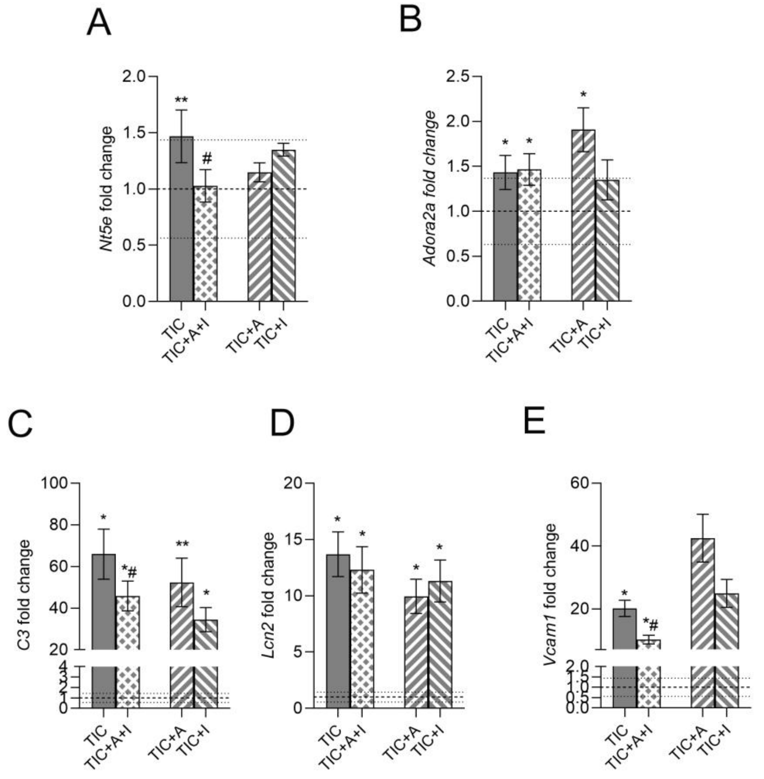

Legend to Supplementary Figure 2.

Combined and single-agent effects of APCP and istradefylline on gene expression in TIC-induced neurotoxic reactive astrocytes.

Gene expression analysis of (A) *Nt5e*, (B) *Adora2a*, (C) *C3*, (D) *Vcam1* and (E) *Lcn2* in TIC-stimulated astrocyte cultures, following combined (TIC+A+I)) or individual treatment with APCP (TIC+A) and istradefylline (TIC+I). Bars represent relative mRNA expression levels (fold change ± SEM) obtained from ≥ 2 independent astrocyte cultures analyzed in technical duplicates. Statistical significance is indicated within the graphs: *p < 0.05 vs. control; **p < 0.01 vs. control; #p < 0.05 vs. TIC.

2 **1.3. Supplementary Figure 3**

Supplementary Figure 3.

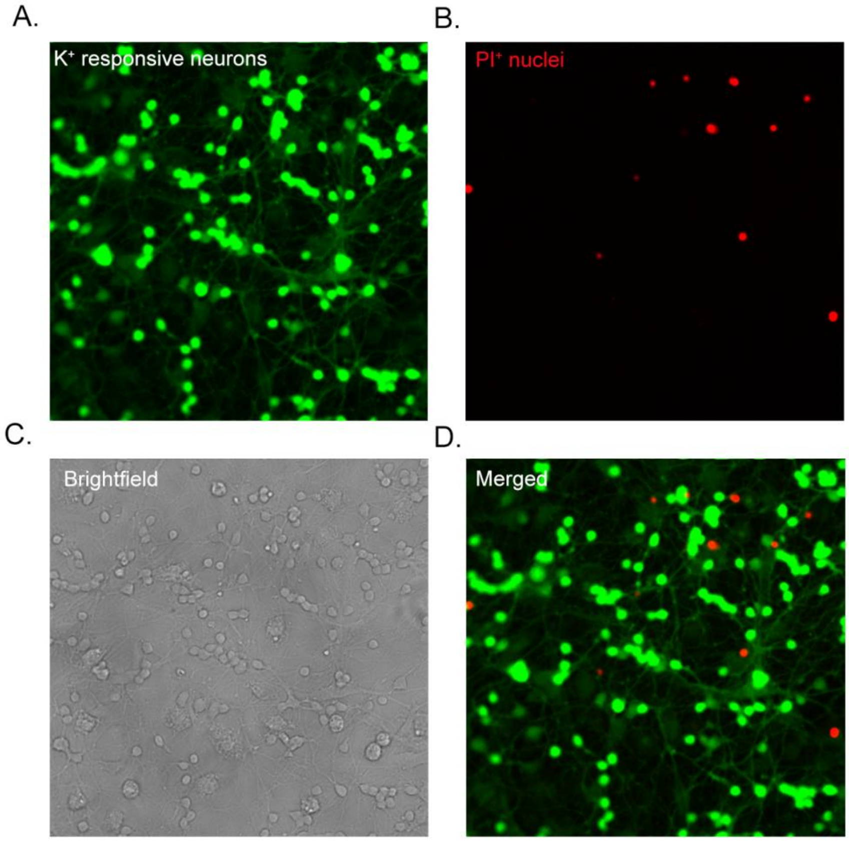

Legend to Supplementary Figure 3.

Identification of of propidium iodide (PI)-positive cells within neuronal population

Comparison of bright-field images and overlaid fluorescence micrographs showing live neurons identified by robust depolarization-induced Ca^{2+} responses (*green fluorescence*) together with PI-positive nuclei (*red fluorescence*) within the same microscopic field.
